# The rise to dominance of genetic model organisms and the decline of curiosity-driven organismal research

**DOI:** 10.1371/journal.pone.0243088

**Published:** 2020-12-01

**Authors:** Sarah M. Farris

**Affiliations:** Department of Biology, West Virginia University, Morgantown, West Virginia, United States of America; University of Leicester, UNITED KINGDOM

## Abstract

Curiosity-driven, basic biological research “…performed without thought of practical ends…” establishes fundamental conceptual frameworks for future technological and medical breakthroughs. Traditionally, curiosity-driven research in biological sciences has utilized experimental organisms chosen for their tractability and suitability for studying the question of interest. This approach leverages the diversity of life to uncover working solutions (adaptations) to problems encountered by living things, and evolutionary context as to the extent to which these solutions may be generalized to other species. Despite the well-documented success of this approach, funding portfolios of United States granting agencies are increasingly filled with studies on a few species for which cutting-edge molecular tools are available (genetic model organisms). While this narrow focus may be justified for biomedically-focused funding bodies such as the National Institutes of Health, it is critical that robust federal support for curiosity-driven research using diverse experimental organisms be maintained by agencies such as the National Science Foundation. Using the disciplines of neurobiology and behavioral research as an example, this study finds that NSF grant awards have declined in association with a decrease in the proportion of grants funded for experimental, rather than genetic model organism research. The decline in use of experimental organisms in the literature mirrors but predates the shift grant funding. Today’s dominance of genetic model organisms was thus initiated by researchers themselves and/or by publication peer review and editorial preferences, and was further reinforced by pressure from granting agencies, academic employers, and the scientific community.

## Introduction

In his historic report to President Franklin D. Roosevelt titled “Science the Endless Frontier,” Vannevar Bush wrote persuasively of the necessity of basic, curiosity -driven research for establishing the foundation of scientific progress [1]. Bush defined basic research as that “… performed without thought of practical ends…. (resulting) in general knowledge and an understanding of nature and its laws” characterized by “…free, untrammeled study of nature, in the directions and by the methods suggested by (the researcher’s) interests, curiosity, and imagination.” Bush argued that such research is essential for the national welfare as “It creates the fund from which the practical applications of knowledge must be drawn…new products and processes…are founded on new principles and conceptions, which in turn are painstakingly developed by research in the purest realms of science.” He concluded that basic research was so important for the continued welfare and security of the United States that it merited federal financial support. “Science, the Endless Frontier” fueled an ongoing debate over the role of government in supporting scientific research, culminating in the establishment of the National Science Foundation (NSF) in 1950 whose mission is, in part, “…to initiate and support basic scientific research… [2, 3].”

Bush [1] cited Alexander Fleming’s discovery of penicillin in a fungal contaminant of bacterial colonies as an example of the process by which an unanticipated, serendipitous observation can initiate a cascade of curiosity-driven then applied research culminating in a medical breakthrough. Curiosity-driven biological research continues to drive advances in technology and medicine [4–7], and the impact of this approach and the need for federal support is appreciated by the majority of the American public [8].

The history of antibiotic development, including Fleming’s Nobel Prize-winning research, also illustrates the invaluable role that the diversity of life plays in generating novel discoveries that drive innovation. The penicillin-producing mold discovered by Fleming, *Penicillium notatum*, did not produce enough of the antibiotic for clinical use, but another serendipitous discovery revealed that a close relative, *P*. *chrysogeum*, was able to produce much larger amounts of the drug [9]. Subsequently, development of a screening assay based on the observation that soil bacteria could inhibit growth of other microbes allowed identification of several antibiotic compounds that are still in clinical use [10].

As these examples illustrate, species-specific adaptations are evolutionary solutions to a problem (competition with bacteria) that can be applied to similar problems in other organisms (bacterial infection). In other cases, the common ancestry of life on earth allows biological processes and mechanisms uncovered in diverse but experimentally tractable species to be generalized to other, more difficult to study organisms, including humans. For example, early research in organisms chosen for their relatively simple neural circuits and behaviors (such the sea slug *Aplysia californica*), uncovered fundamental and universal properties of learning and memory (reviewed in [11]).

Modern neuroscience rests on a foundation of discoveries made in evolutionarily and phylogenetically diverse species. The immense value of this approach is reflected by the diversity of animal models in Nobel Prize-winning neuroscience research. These breakthroughs include Ramon y Cajal’s and Sherringtons’s comparative neuroanatomy and physiology [12, 13]; the work of Adrian, Eccles, Hodgkin and Huxley on the electrical activity of neurons in frog, earthworm and squid [14–16]; that of Keffer Hartline on early visual processing in the horseshoe crab [17]; and the above-mentioned work of Eric Kandel on the neural basis of learning and memory in the sea slug *Aplysia*.

A robust example of the value of experimental organism diversity in biological sciences in general and neuroscience specifically is observed in the field of neuroethology (the study of the neural basis of natural behavior). Neuroethology leverages experimental organism choice so as “…to take advantage of evolutionary solutions to biological problems to identify both behavioral adaptations and genetic mechanisms [18].” Like “traditional” neuroscience, neuroethological model organisms are chosen for their experimental tractability and suitability for studying the question of interest, but additionally for their natural behaviors that are particularly well adapted to solve the problem of interest [19, 20]. When combined with comparative neuroanatomy, fundamental principles of nervous system structure and function emerge, while species diversity illuminates the roles of novelty and constraint on adaptive solutions to problems encountered by animals in their environment [21]. Following the terminology of Ankeny [22], species chosen for research in this manner are hereafter referred to as “experimental organisms” (EOs).

In contrast to its historical breadth, modern neuroscience research is heavily focused on a small number of species that are considered models for biological processes that are considered generalizable across taxa. Again following the terminology of Ankeny [22], these species are here referred to as “model organisms” (MOs). These species were initially adopted due to their suitability for laboratory rearing and use in research requiring rapid life cycles, high fecundity, and environmental and genetic homogeneity (e.g. development and genetics). The basic biology of these species was characterized by communities of scientists building knowledge bases and research infrastructure that promoted development of molecular genetic experimental tools [22–25]. Later, the development of gene transformation technology in four of these species (the mouse *Mus musculus*, zebrafish *Danio rerio*, fruit fly *Drosophila melanogaster*, and nematode *Caenorhabditis elegans*, hereby referred to as genetic model organisms, or GMOs, positioned researchers to exploit the insight generated by the Human Genome Project [26, 27]. In 1999, the National Institutes of Health (NIH; the primary biomedical funding agency in the United States) published a “canonical list” of model organisms including the aforementioned four species and three additional species, the African clawed frog *Xenopus laevis*/*tropicalis*, the domestic chicken *Gallus gallus*, and the rat *Rattus norvegicus* (the original 1999 online publication “Non-mammalian models workshop” is no longer available online, but the list of organisms cited can be found in studies by Ankeny [23] and Dietrich et al [28]. Shortly afterwards, the announcement of the NIH Roadmap [29, 30] emphasizing translational (“bench to bedside”) research ushered in the current era of genetic model organism dominance in biomedical research. The newly realized scope of genome conservation across animals meant that mechanistic studies in these species, especially those with genetic transformation tools, meant that a plausible case could be made for the potential of genetic model organism research to translate to human health, thus attracting funding from the National Institutes of Health.

The highly focused GMO approach has been enormously successful: in the field of neuroscience, and nervous system structure and function has been dissected at unprecedented resolution [31–33]. However, there is increasing concern that the flood of GMO research driven by ever-advancing molecular genetic methods has come at the expense of curiosity-driven basic research including that using EOs [23, 28, 34–44]. These concerns are supported by studies showing shifts in species usage in NIH-funded grants and publications [28, 42], and pressure (either real or perceived) for prospective NIH grantees to focus on GMO research with plausible applicability to human health [45–53]. Whatever the cause, a substantial decline in curiosity driven EO research that has been such an essential component of scientific breakthroughs is likely to have dire consequences for scientific progress.

As previously described, the National Science Foundation (NSF) is a critically important source of dedicated funding for curiosity-driven research, “the fund from which the practical applications of knowledge must be drawn [1].” Regardless of the state of NIH funding, the explicit mission of the NSF should maintain robust support for research without obvious applications, utilizing both EOs and GMOs where appropriate. This study seeks to determine whether the NSF is indeed fulfilling this role by analyzing funding and publications for curiosity-driven research in both GMOs and EOs. Due to the large amount of available funding data, this analysis is focused fields of neuroscience and behavior (NBR) which includes all types of neuroscience from highly applied to basic research exemplified by the discipline of neuroethology. The latter particularly serves the purpose of this study as is has a long history of robust NSF support and publication in the primary literature, and characteristically employs a wide range of species including GMOs. NIH funding for EO and GMO NBR is also calculated for comparison with NSF funding during the same time period.

## Materials and methods

### Neurobiological and behavioral research funding

The NSF Award Search advanced search engine (https://www.nsf.gov/awardsearch/advancedSearch.jsp) was used to gather award data from the BIO Directorate, the Division of Integrative Biology and Neuroscience (IBN; 1987–2004), Division of Integrative and Organismal Biology (IOB; 2004–2007), and the Division of Integrative Organismal Systems (IOS; 2007-present) every other fiscal year (Oct 1- Sept 30) from 1987–2017. From FY 1987–2004, the IBN Neuroscience cluster was composed of the Behavioral Neuroscience, Computational Neuroscience, Developmental Neuroscience, Neuroendocrinology, Neuronal and Glial Mechanisms, and Sensory Systems programs. The Animal Behavior program was contained within the Physiology and Ethology cluster. From 2004–2007, neuroscience-focused programs at NSF were combined to form the Environmental & Structural Systems and Functional and Regulatory Systems clusters. The Animal Behavior program was placed within the Behavioral Systems Cluster where it has remained through the present. In 2007, a single Neural Systems cluster was established and subdivided into the Organization, Activation, and Modulation programs which remain today. It should be noted that the transition between IBN, IOB, and IOS clusters overlapped; for example, an award granted in 2007 might be made through both the older Environmental and Structural Systems Cluster and the Activation program (part of the newer Neural Systems Cluster). Documentation of the organization of these divisions and clusters was obtained from the NSF Document Library (https://www.nsf.gov/publications/). Training, conference, and travel awards were removed from the total award data so that only research awards were considered in subsequent analyses. In addition, each research project was counted as an award; for example, multi-PI grants and those designated as “Collaborative Research” were each counted as a single “award” as funding was for the same research project.

Neurobiological and behavioral research (NBR) award amounts were converted to 2018 United States dollars ($) using the CPI Inflation Calculator (United States Bureau of Labor Statistics (https://data.bls.gov/cgi-bin/cpicalc.pl)). Calculations of award dollars per PI on collaborative awards were calculated from the total dollars disbursed via multi-PI and “Collaborative Research” awards divided by the total number of PIs on these grants. The NSF grant database provides dollar amounts awarded to date; total funds awarded are available only for expired awards. Thus, grant funding could only be considered through 2011, the last year for which all awards had been completed at the time data was collected. Total numbers of grants awarded and PIs funded could be analyzed up to 2017. The number of proposals and preliminary proposals submitted to and funded by NBR clusters were not available, even upon FOIA request. However, this data was available for IOS from 2001–2018 at https://dellweb.bfa.nsf.gov/ and https://catalog.data.gov/dataset/nsf-research-grant-funding-rate-history. The number of preliminary proposals submitted to IOS from 2013–2015 was obtained from [54].

Model organisms used in funded NSF NBR grants were quantified via a curated search of abstracts for each funded NBR award and, if not stated, from a PubMed search (https://www.ncbi.nlm.nih.gov/pubmed) of publications by the PI at the time of the award. Model organisms are subdivided into two categories. The first, genetic model organisms (GMOs), are defined as animals for which molecular tools exist for germ-line transformation and modification of gene expression. These animals are included on the list of “canonical” model organisms created by the National Institutes of Health in a 1999 online publication titled “Non-mammalian models workshop.” This document is no longer available online, but studies by Ankeny and Dietrich et al [23, 28] provide the list of organisms. Organisms considered GMOs are the roundworm *Caenorhabditis elegans*, the fruit fly *Drosophila melanogaster*, the mouse *Mus musculus*, and the zebrafish *Danio rerio*. All other animal species used in funded research, as well as computational research, were grouped as experimental (non-genetic) organisms (EOs). Note that EOs include some species on the 1999 canonical model organisms list that lack molecular tools (the chicken *Gallus gallus*, the African clawed frog *Xenopus laevis* or *X*. *tropicalis*, and the rat *Rattus rattus*).

The NIH RePORT search engine (https://report.nih.gov/index.aspx) was used to collect data on research project grants (RPGs) awarded by NIH neuroscience programs every other year from 1995–2017. Institutes participating in the NIH BRAIN Initiative were included in the search [55]. Awards were searched by the 7 model organisms listed in the previous paragraph.

### Neurobiological and behavioral research publications

A Web of Science advanced search (https://apps.webofknowledge.com) was used to identify journal articles published in the Neurosciences subject area by year, model organism, and United States authorship. First, curated Web of Science topic searches were performed from 1990–2017 for comparisons of taxonomically related GMO:EO pairs, the fruit fly *Drosophila melanogaster* vs. insects (all other insect species) and the zebrafish *Danio rerio* vs. fish (all other fish species). A second publication search of model organisms on the NIH “canonical model” list (from 1995–2017 to match the time period for which NIH award data was collected) was tabulated using only article titles. Title searches were chosen for this analysis as topic searches for these popular model species produced hundreds or thousands of “hits” that preclude curation of results. Title searches underreport model species usage as the species is not always stated in the article title. However, topic searches over-report model species usage (for example when related findings in mouse are described in the abstract of a study performed using rats). To demonstrate the degree to which overcounting and undercounting occur in topic and title searches, the first 100 hits of the topic search for the mouse model species in the years 2005, 2011, and 2017 were curated. Both over and undercounting occurred, but at similar levels in all three years (13, 14, and 17 overcounts and 36, 38, and 39 undercounts for 2005, 2011, and 2017 respectively). Since both errors appeared consistent across the three dates, title search provides a stable representation of publication trends by model species.

### Data analysis

Data was plotted using Microsoft^®^ Excel^®^ for Mac 2011 (Microsoft Corporation, Redmond WA, USA) and fit of regression lines was determined via ANOVA using the GraphPad QuickCalcs browser application (GraphPad Software, San Diego CA, USA) (https://www.graphpad.com/quickcalcs/). Figures were constructed using Microsoft^®^ Powerpoint^®^ for Mac 2011 (Microsoft Corporation, Redmond WA, USA).

## Results and discussion

### Neurobiological and behavioral research funding by the National Science Foundation

**Fig 1:**
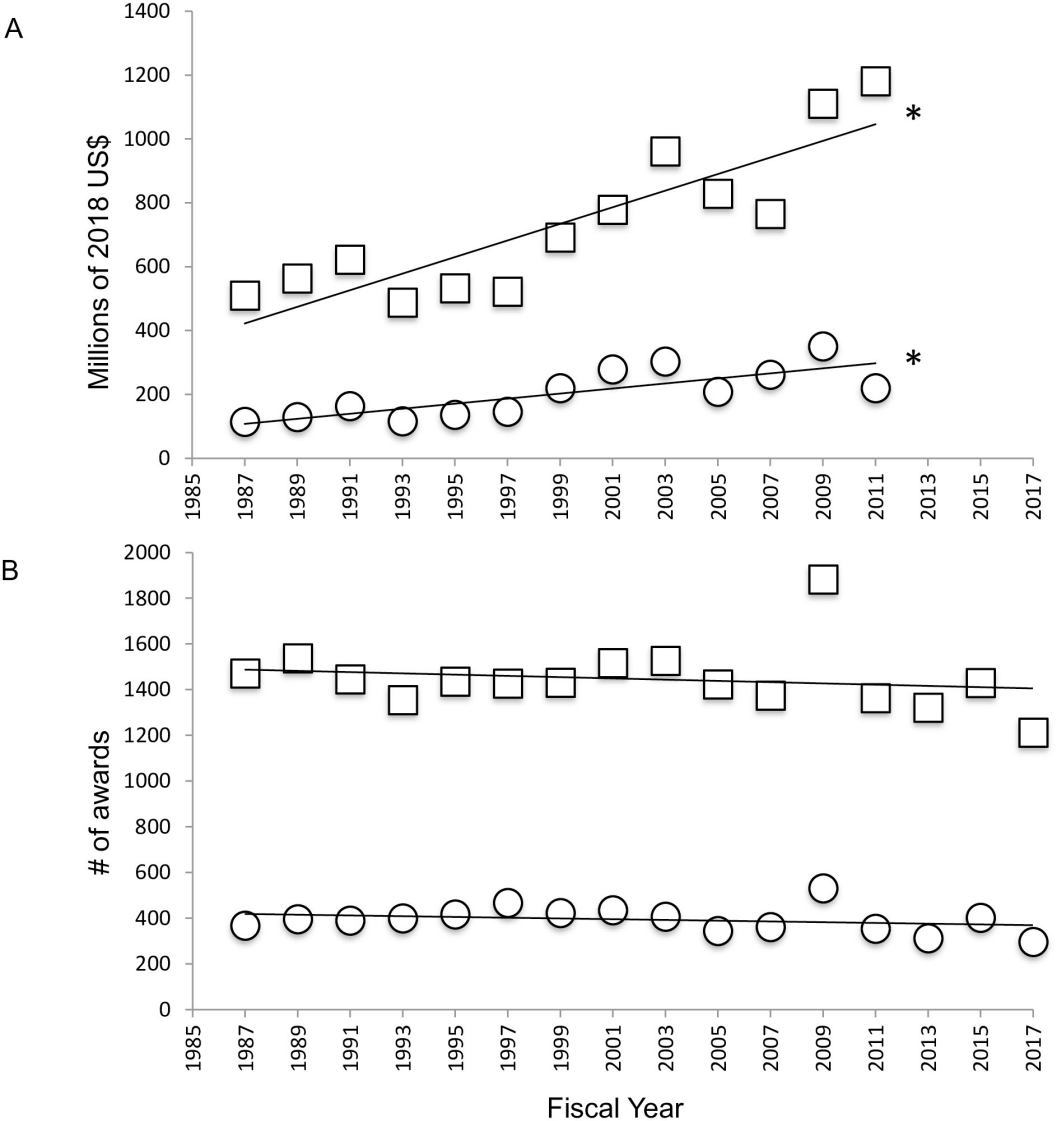
Grant funding by NSF BIO and IOS. A. Total 2018 US$ awarded every other year from FY1987- FY2011 by the BIO directorate (squares) and IOS division (circles). B. Total award numbers made every other year from FY1987- FY2017 by the BIO directorate (squares) and IOS division (circles). Asterisks in A indicate significant increase in funded grant dollars for both BIO and IOS.

Neuroscience and behavior research (NBR) is funded by the NSF under the Division of Integrative and Organismal Systems (IOS; previously designated the Division of Integrative Biology and Neuroscience or the Division of Integrative and Organismal Biology) that is in turn contained within the BIO directorate. Funding through these bodies was determined via an advanced award search for every other fiscal year from 1987–2011, after which total funds disbursed was not available for ongoing awards. When converted to 2018 US dollars, BIO funding (Fig 1A; F(1, 11) = 35.78, p<0.0001, R^2^ = 0.7648) and IOS funding (Fig 1A; F(1,11) = 19.05, p = 0.0011, R^2^ = 0.6340) is observed to have increased significantly. From 1987–2017 the total number of awards made by both the BIO directorate (Fig 1B; F(1, 14) = 0.4856, p = 0.4973, R^2^ = 0.0335) and the IOS division (Fig 1B; F(1, 14) = 1.113, p = 0.3094, R^2^ = 0.0736) remained constant. Thus, on average, the dollar amount per award increased from 1987–2011 for grants made through the BIO directorate and the IOS division.

**Fig 2:**
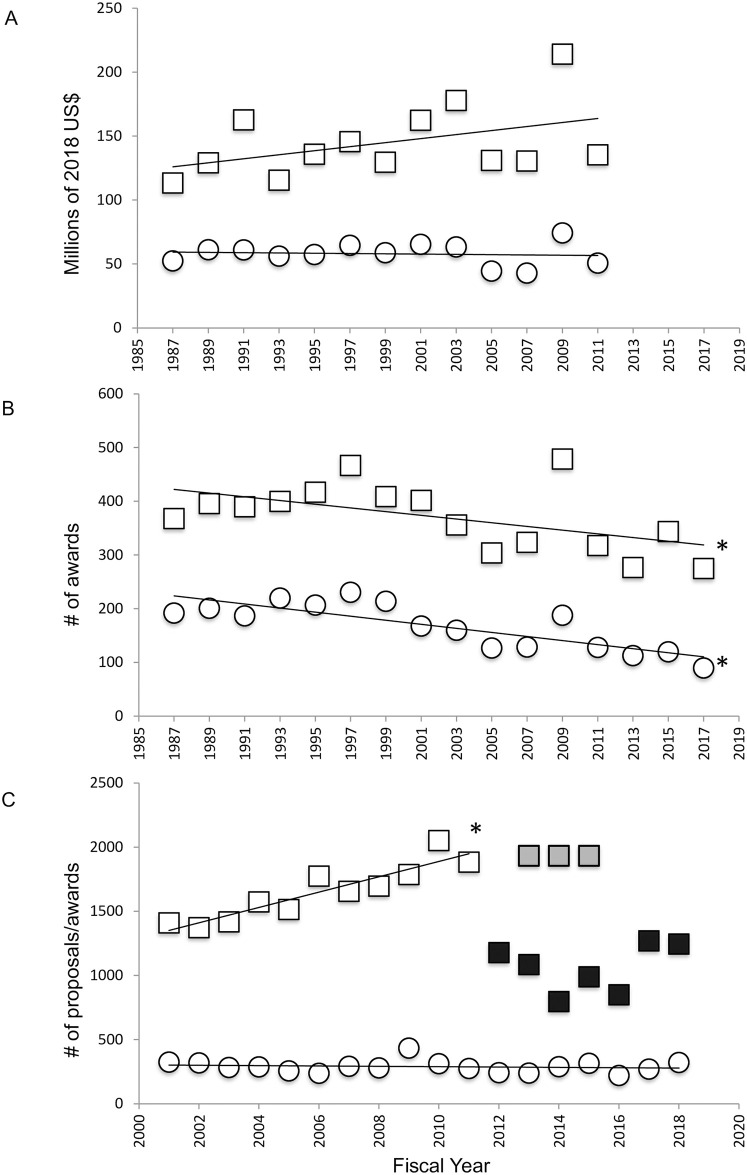
Grant funding by NSF IOS and by neurobiology and behavior programs, and grant success rates for IOS. A. Total 2018 US$ awarded every other year from FY1987- FY2011 by the IOS division (Plant Genome Project awards subtracted (IOS-PGP); squares), and neurobiological and behavioral (NBR) programs contained within IOS (circles). B. Total award numbers made every other year from FY1987- FY2017 by IOS-PGP (squares) and NBR programs (circles). C. Full proposals submitted to IOS from 2001–2011 (open squares), estimated preproposals submitted from 2013–2015 (light grey squares), invited full proposals submitted from 2012–2018 (black squares) and total grants funded from 2000–2018 (open circles). Asterisks in B indicate significant decrease in IOS-PGP and NBR award numbers. Asterisk in C indicates significant increase in IOS proposals submitted.

Beginning in 1998, grants funded through the Plant Genome Project (PGP) were added to the IOS Division [56]. When PGP grants are subtracted from IOS (IOS-PGP), the amount of funding disbursed via IOS was unchanged from 1987–2011 (Fig 2A; F(1, 11) = 2.635, p = 0.1328, R^2^ = 0.1933) and the number of awards made decreased (Fig 2B; F(1, 14) = 5.736, p = 0.0312, R^2^ = 0.2906). NBR funding mirrored that for IOS-PGP (Fig 2A; F(1, 11) = 0.1099, p = 0.7465, R^2^ = 0.0099; Fig 2B; F(1, 14) = 28.29, p = 0.0001, R^2^ = 0.6690). As observed for BIO and IOS, IOS-PGP and NBR awards were fewer and larger; but resulted from a decline in award number under a flat budget.

Do decreasing numbers of awards funded reflect a decrease in proposals submitted? While proposal submission data for NBR or IOS-PGP was not available, data for IOS revealed a steady increase in proposal submissions from 2001–2011 (open squares, Fig 2C; F(1, 9) = 49.87, p<0.0001, R^2^ = 0.8471), while the number of grants awarded remained unchanged (open circles, Fig 2C; F(1, 16) = 0.3850, p = 0.5437, R^2^ = 0.0235), which would result in a decline in funding success rate. In 2012 IOS adopted a preliminary proposal requirement [57], from which a portion were chosen for full proposal submission. Katz et al [54] reports that from 2013–2015 a total of 5802 preproposals were submitted to IOS; divided equally across the three-year period, the number of preproposals submitted per year during this period was similar to the number of proposals submitted in 2012 (light grey squares, Fig 2C). Katz et al [54] and two NSF funding databases (see Materials and methods) report the number or preproposals selected for invited submission as full proposals (black squares). Under the preproposal system, the absolute number of grants funded (open circles) did not differ from that prior to implementation of the preproposal requirement. Although the data in Fig 2C is for the entirety of IOS and not just NBR programs, it suggests that the observed decrease in IOS-PGP and NBR grants funded is unlikely to have resulted from a decrease in submitted proposals.

**Fig 3:**
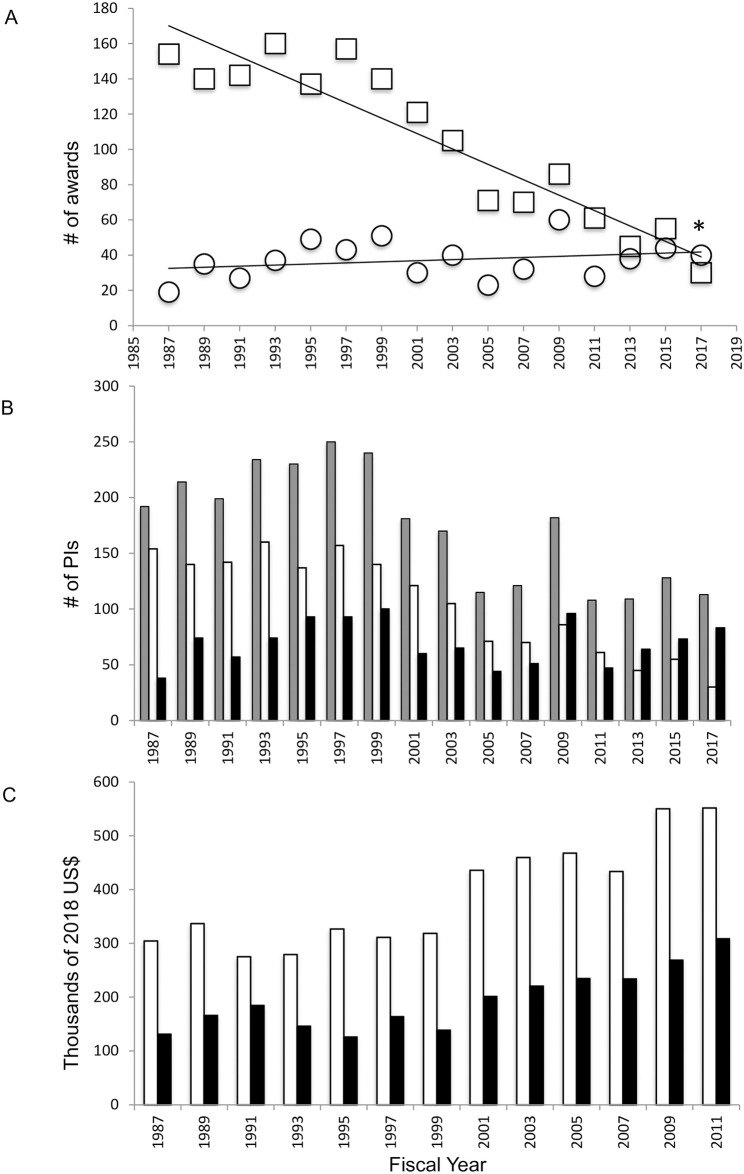
Grant funding by NSF NBR programs for collaborative and sole PI projects. A. Sole PI (squares) and collaborative (circles) NBR awards funded from FY1987- FY2017. B. Total PIs funded (grey), sole PIs (white) and individual PIs funded on collaborative grants (black). C. Mean award amounts for sole PIs (white) and PIs on collaborative grants (black). Collaborative project dollars per PI were calculated as the total amount of dollars awarded by collaborative grants divided by the total number of PIs funded by those grants. Asterisk in A indicates significant decrease in funded sole PI grants.

Declining awards for NBR particularly impacted those for sole PI research (excluding conference, travel, and dissertation grants; Fig 3A; F(1, 14) = 98.01, p<0.0001, R^2^ = 0.8750). The number of sole PI awards was relatively constant from 1987–1999, peaking at 160 funded grants in 1993. After 1999, sole PI awards dropped precipitously such that only 30 were granted in 2017, a decline of over 80%. In contrast, no change was observed in the number of collaborative projects funded from 1987–2017. These included grants specifically designated as “Collaborative Research” and disbursed as separate funds to each PI, and multi-PI projects awarded as lump sums (Fig 3A; F(1, 14) = 1.120, p = 0.3078, R^2^ = 0.0741 Although the number of collaborative grants made remained constant, an increasing proportion of researchers were funded by these awards (Fig 3B). On average, PIs on collaborative grants (determined by dividing total funding disbursed by these awards by the total number of collaborative PIs) received smaller dollar amounts than did sole PIs (Fig 3C). To summarize, when compared with funding data from 30 years the today’s NSF NBR programs fund fewer PIs with smaller awards on average.

### Model organism usage in NBR NSF and NIH awards and publications

**Fig 4:**
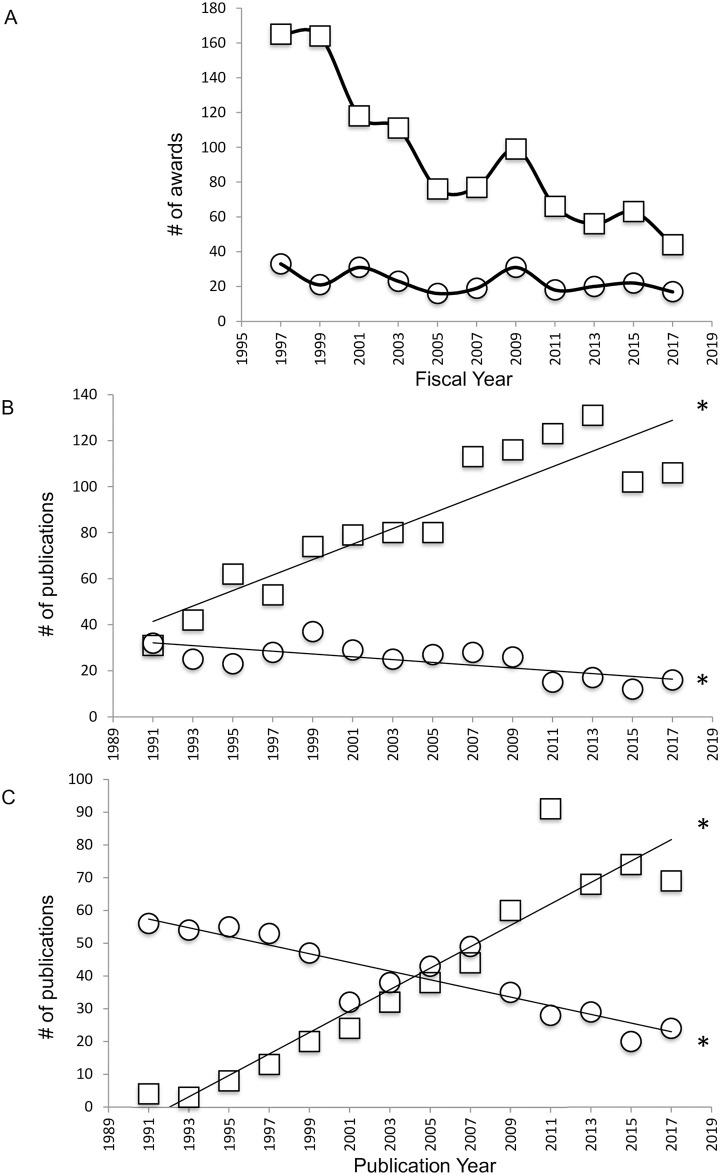
Publications for GMO and EO species within the insects and fish (Chondrichthyes + Osteichthyes). A. NSF neuroethology awards for non-genetic model species research (EOs, squares) and genetic model species research (GMOs, circles) from 1997–2017. B. Curated publications by United States authors using the *Drosophila melanogaster* model species (squares) and other insect model species (circles). C. Curated publications by US authors using the zebrafish model species (squares), and other fish model species (circles), Asterisks in B indicate significant increase in *Drosophila* publications (top) and decrease in other insect publications. Asterisks in C indicate significant increase in zebrafish publications (top) and decrease in other fish publications.

Declining NSF NBR grant funding particularly impacted the number of grants awarded for research using EOs (Fig 4A; F(1, 9) = 49.13, p<0.0001, R^2^ = 0.8452). In contrast, award numbers for GMO research remained low but constant from 1997–2017 (Fig 4A; F(1, 9) = 3.286, p = 0.1033, R^2^ = 0.2675). This resulted in proportionally larger numbers of grants awarded for GMO research; almost 28% of NBR research awards in 2017 compared with 17% in 1997.

Declining numbers of NBR publications using EOs is associated with but predates decreasing grant funding for EO research (by both the NSF and NIH, as shown below). Two curated Web of Science topic searches of US authored NBR publications compared large phylogenetic taxa (insects and fish) with genetic model organisms contained within those taxa (*Drosophila melanogaster* and *Danio rerio*, respectively). Research articles for EO insect or fish species have significantly declined (Fig 4B insects, F(1,12) = 13.04, p = 0.0036, R^2^ = 0.5209; Fig 4C fish, F(1, 12) = 46.77, p<0.0001, R^2^ = 0.7958) while publications for GMOs within those groups have significantly increased (Fig 4B *Drosophila*, F(1, 12) = 54.00, p<0.0001, R^2^ = 0.8182; Fig 4C *Danio*, F(1, 12) = 98.76, p<0.0001, R^2^ = 0.8917).

**Fig 5:**
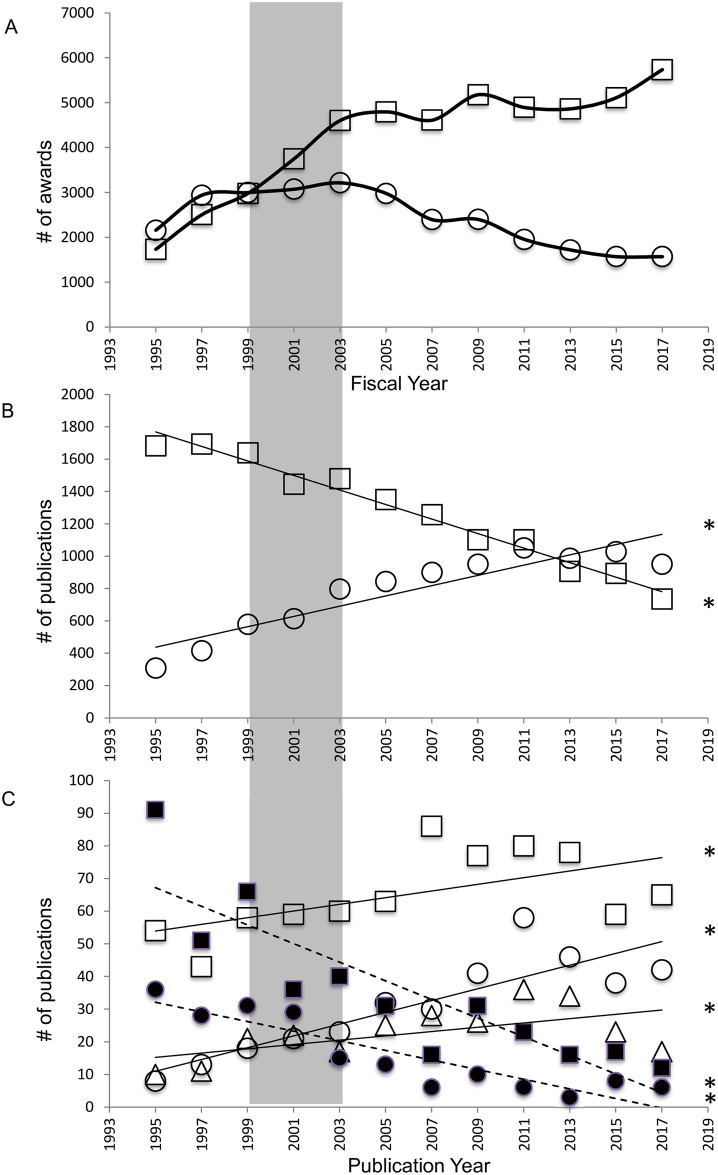
NIH grant funding and publications for GMOs and MOs on the 1999 canonical model species list. A. NIH neuroscience awards for canonical animal model organisms with (squares; GMOs) and without (circles; MOs) molecular genetic tools. B. Title search for publications using the canonical rodent model species rat (squares; MO) and mouse (circles; GMO). C. Title search for publications using canonical GMOs (*Drosophila* (open squares), zebrafish (open circles), *C*. *elegans* (open triangles)) and canonical MOs (chick (closed squares), *Xenopus* (closed circles)). Shaded area indicates time period between release of the NIH canonical model species list and the NIH Roadmap. Asterisks indicate significant increase in publications using *Drosophila*, zebrafish, and *C*. *elegans*, and significant decrease in publications using chick and *Xenopus*.

NIH RePORTER was used to search for NIH research awards using the canonical model species (both genetic (GMOs) and non-genetic model organisms (MOs)) by NBR-focused institutes. While awards for both GMO and MO research appeared to be roughly even prior to 1999, those for GMOs increased steadily from 1995–2003 (prior to and including the time period between release of the canonical organism list and the NIH Roadmap, as indicated by the grey box in Fig 5A). Awards for MO remained were steady from 1997–2003, after which they began to decline (Fig 5A). After 2003, MO NBR awards declined overall while those for GMOs steadily increased. Web of Science publication searches by date, model organism, and US authorship (Fig 5B and 5C) revealed increasing numbers of publications for GMOs on the canonical organisms list (mouse, F(1,10) = 56.27, p<0.0001, R^2^ = 0.8491; *C*. *elegans*, F(1, 10) = 5.283, p = 0.0444, R^2^ = 0.3457; *Drosophila melanogaster*, F(1,10) = 5.242, p = 0.0450, R^2^ = 0.3439; and *Danio rerio*, F(1,10) = 33.87, p<0.0002, R^2^ = 0.7721) and decreasing numbers of publications for non-genetic model species (rat, F(1,10) = 395.2, p<0.0001, R^2^ = 0.9753; *Xenopus*, F(1,10) = 45.26, p<0.0001, R^2^ = 0.8190; and chick, F(1,10) = 31.78, p<0.0002, R^2^ = 0.7606). As with the curated search, these trends began prior to the 1999–2003 time period (grey box, Fig 5) during which the NIH canonical model organisms list and NIH Roadmap were released.

The steady decline of curiosity-driven research in the US, particularly that with the broad organismal and evolutionary scope facilitated by use of EOs, has been decried by many authors [23, 28, 34–44]. The present study sought to empirically characterize the 30-year course of curiosity-driven neuroscience and behavioral research (NBR), particularly that using EOs, as measured by grant funding and publications. NBR funding by the NSF, whose mission prescribes support for curiosity-driven research, was compared with that by the NIH, which invests in research relevant to human health and disease. It was expected that in the face of growing emphasis on “translational” research at the NIH, facilitated by use of GMOs, the NSF would continue to serve as a bastion of crucial support for EO, curiosity driven NBR. Instead, the NSF award data presented above describes a clear and alarming decline in the number of grants funded for NBR and organismal biology as a whole (IOS; excepting that allocated for the Plant Genome Project). From a peak total of 200 NBR grants awarded in 1997 (not including training, meeting, and travel awards, and those awarded from the 2009 ARRA), only 70 grants were funded in 2017, a 65% decrease (Fig 2B). The number of PIs funded for NBR also declined from a peak of 250 in 1997 to 113 in 2017, a 55% decrease (Fig 3B). Data available for IOS from 2001–2015 demonstrates that proposal submissions have increased while the number of grants funded has remained unchanged, suggesting that a decline in NBR grant success rate may underly the deterioration of NBR support (Fig 2C). The casualties of declining NBR grant funding have overwhelmingly been awards to sole PIs and for NBR utilizing EOs (Figs 3A and 4A), such that awarded grants fund proportionally more genetic model organism research and more PIs on collaborative grants (Fig 3B). It is important to note that collaborative grants per PI are on average significantly smaller than those for sole PIs: in 2017, the mean award amount for sole PI NBR grants was $551, 861 over 2–5 years, while that for a PI on a collaborative grant was on average $308, 593 over 2–4 years, just 56% of that for sole PIs (in 2018 dollars; Fig 3C). It is also important to consider that these amounts represent *total* dollars awarded. The NIH “bread and butter” R01 grant lasts up to 5 years and is funded for up to $250,000 in *direct* costs per year (if the proposal uses a simplified modular budget; far larger budgets are allowable with additional justification [58]) Finally, as observed for NSF funding of EO NBR, NIH funding for NBR using EOs on the “canonical model organism” list has declined, although in contrast to the NSF, NIH awards for GMO NBR have increased (Fig 4A).

Dwindling funding support for EO NBR is mirrored by declining publications for such research. Analysis of NBR publications reveals that research on EOs, is essentially being replaced by that utilizing a handful of GMOs. In 2017, NBR publications for single GMOs (e.g. *Drosophila* or zebrafish) greatly outnumbered those for the entire taxa those species belong to (e.g. insects or fish; Fig 4B and 4C). As an example, 28 NBR studies were published by US labs on insects excluding *Drosophila* in 1997 and 16 were published in 2017. In contrast, *Drosophila* publications numbered 53 in 1997 (65% of total papers on insect NBR) and 106 in 2017 (87% of insect NBR papers). Species included in the 1999 NIH canonical list of species that lack the advanced technology for genetic manipulation and are thus similar to EOs (e.g. rat, *Xenopus*, chick) have also declined in representation in the literature (Fig 5B and 5C). These once popular and well-supported model systems have also fallen out of favor with NBR scientists as GMOs have risen to dominance.

Similar publication trends for GMOs and EOs on the NIH canonical species list were observed for countries outside of the US; GMO publications increased, while EO publications decreased (S1 Dataset). This further supports the hypothesis that US funding bodies are not entirely responsible for the shift towards GMO NBR. Interestingly, curated topic searches revealed that while non-GMO fish and non-GMO insect research publications declined in the US, no significant decline was observed in other countries (other countries, non-GMO fish, F(1,12) = 4.142, p = 0.0645, R^2^ = 0.2566; other countries, non-GMO insects, F(1,12) = 0.6635, p = 0.4312, R^2^ = 0.0524; S1 Dataset, S1 Fig). Although it is difficult to determine why this is the case, it would be interesting to consider whether funding bodies in other countries are currently more amenable to curiosity-driven, non-GMO research, or whether other pressures to focus on more applied, GMO NBR differ outside of the United States.

It is important to note that decreased representation of EOs in US-authored NBR publications predates the shift in funding by both the NSF and the NIH. Changing funding priorities did not drive the initial movement of the scientific community to GMOs but rather appears to have been initiated by researchers themselves. Why did this occur, when the traditionally diverse model species approach had been so successful? The 1980s-1990s was a period of rapid development and refinement of key molecular tools such as targeted recombination, germ line transformation, and exogenous control of gene expression for three established laboratory organisms (*Drosophila*, *C*. *elegans*, and *mouse*), and the introduction of the zebrafish as vertebrate developmental model system ([59–68]; see [24] for review). Later, publication of genome sequences for these GMOs associated with the Human Genome Project cemented these species’ importance as biological research models [26]. Published in top-tier scientific journals, these technologies generated tremendous excitement as they heralded breakthrough approaches and insights. The extent of gene conservation across taxa revealed by genome sequences and the ability to unravel gene functions at unprecedented resolution made GMOs attractive to new and established researchers [23, 44]. In particular, gene conservation with humans made a convincing case for the applicability of GMO research to human health and disease and thus access to research dollars from the largest science funding agency in the United States. NIH administration has consistently maintained that despite the emphasis on translational research, curiosity-driven research using EOs is valued and supported [46–53]. However, these statements are at odds with the experiences of researchers suggesting that the peer review process appears to favor proposals focused on plausible translational outcomes using cutting edge molecular genetic tools available in GMOs [41, 44, 45, 49–51, 69–71]. Under the pressure of a highly competitive grant funding and academic employment climate, researchers will eschew curiosity-driven and EO research in favor of GMOs and projects that can be tailored to current funding trends and publication peer-review and editorial preferences. Alternately, they may shape their research interests around the capabilities of a GMO rather than choosing the model organism most suitable for the intended research. Under each of these scenarios, questions that cannot be adapted to study in GMOs may simply not be pursued [18, 22, 25, 39, 41, 44, 72].

The forces driving declining support for EO NBR by the NSF are less clear but have resulted in an overall reduction in funded PIs and projects despite total expenditures per year remaining unchanged. As observed for NIH grants, the proportions of NSF awards for GMO vs EO NBR began to shift around the year 2000 but were due entirely to reduced support for EO proposals without a concomitant increase in support for GMO grants. In contrast, this shift should not have been influenced by changes in NIH funding priorities such as the designation of canonical model organisms or emphasis on translational research. While it appears that a dramatic change in NBR funding strategy occurred at the NSF concomitant with a similar shift at the NIH, official administrative policy statements to that effect are lacking.

The case for maintaining robust support for curiosity-driven research was well stated by Vannevar Bush and has been reiterated by many authors since. [1, 6, 34, 73]. The contributions of EOs to scientific progress are also clear. EOs enable discovery and study of processes that may not be experimentally tractable or even present in GMOs, often in organismal, ecological, and evolutionary contexts that are critical for determining the degree of generalization of findings [18, 35, 41, 74]. It is important to note that GMO research has also been undeniably successful, enabling important breakthroughs in both curiosity-driven and applied contexts. But the increasing dominance of an exceedingly small number of GMOs in NBR and other areas risks producing a misleading picture of biological processes that complicates generalization. Many authors have cautioned against over-generalizing findings of GMO research to other species, including humans [18, 22, 23, 25, 35, 39, 41, 43, 44, 72, 75–80]; in short, despite gene conservation, GMOs may not model fundamental biological processes as faithfully as they are purported to. To begin with, GMOs were initially selected for their derived life histories that facilitate laboratory rearing and research (small size, rapid life cycle and development) underpinned by molecular and cellular adaptations that may not be generalizable to species that did not undergo selection for these characteristics [25, 72, 81]. Furthermore, GMO laboratory lines have been “standardized” by years of inbreeding and strict environmental control to remove variation. While this approach facilitates study of biological mechanisms, it is not representative of how these mechanisms operate in a animal living in the natural world, where genetic, phenotypic, and environmental variation is the norm [82–85].

Another limitation to generalization is the fact that genetic homology does not always equate to structural or functional homology; the role of a gene in mouse is not always the same as that in a human. Millions of years of independent selection and adaptation in GMOs scattered across animal phylogeny influences gene expression and function [44, 72, 86]. New functions acquired by ancient genes as a result of regulatory and coding sequence divergence have been extensively documented [87–89] such that genes with conserved coding sequences may have very different functions [90, 91]. Furthermore, convergent traits in different species may appear to be homologous, even though they arose independently and may rely on entirely different genetic mechanisms (reviewed in [72]). Without comparative studies incorporating a range of EOs, it is impossible to know the extent of conservation of a biological process elucidated in GMOs that are a vanishingly small representation of all life on earth.

As may be expected, it is increasingly evident that GMOs are not universally suitable models for all aspects of human health and disease [41, 43, 74, 78, 92]. A particularly clear example of this issue is the failure of hundreds of clinical trials for Alzheimer’s disease treatments that were efficacious in mice (reviewed by [93]). Disease phenotypes are induced in GMOs by manipulation expression of a candidate gene in an otherwise homogenous genotypic and environmental background. As such these laboratory-generated disease models may not consider factors important for the onset and progression of human disease. Attempts to model the neural bases of behavior are similarly limited by the focused precision afforded by GMOs: does the activity of a select population of neurons during a simple behavioral assay in a homogeneous population of a single derived species provide broad insight into general principles of brain function [72, 78–80]? How does a behavior chosen for ease of quantification in the laboratory relate to the natural behavior of the animal in a complex, changing environment? Is the activity of cells and circuits that evolved to enable these adaptive behaviors faithfully recapitulated under laboratory conditions? Once again, the relevance and generalizability of GMO research cannot be fully appreciated outside of a comparative context and without a detailed understanding of how laboratory conditions relate to the natural behavior of the GMO [94–97].

Finally, there is concern that the most attractive aspect of GMO research, the continually developing array of molecular genetic tools, encourages a deeply reductionist approach resulting in an exceedingly narrow view of biological processes [25, 37, 38, 79, 80]. Bolker [39] aptly states: “The extraordinary resolving power of core models comes with the same trade-off as a high-magnification lens: a much-reduced field of view.” While allowing profound insight into genetic and cellular mechanisms, their relevance to the whole organism in its environment and as a member of the animal kingdom can only be conjectured.

Continued scientific progress demands that mechanistic GMO research with prospective applications must be brought back into balance with curiosity-driven research, including that using EOs. A critical step towards restoring balance is to bring the experimental capabilities of selected EOs in line with those of GMOs by providing robust financial support for the development of molecular tools and infrastructure [41, 98]. Federal support for such purposes was instrumental to building GMO scientific communities [22, 23, 25, 78]. Initial efforts in building EO infrastructure might be directed towards refining and standardizing existing molecular genetic tools that are most likely to work in a wide range of species (e.g. CRISPR-Cas9). EOs or groups of EOs should initially be selected based on their phylogenetic position and suitability for studying particular evolutionary or biological processes, by scientific communities united by research interest (e.g. the neuroethology community in the case of NBR [86, 91, 98–101]). Once chosen, it is critical that curiosity-driven investigation of EO biology be coupled with the comparable molecular genetic tools, establishing these species as truly holistic model organisms. Once established, support for EO research and infrastructure must be maintained in the long term as it has been for GMOs, funding proposals targeted towards continued development of EOs and curiosity-driven research using both EOs and GMOs.

The current highly competitive funding climate promotes a fatalistic attitude towards the possibility of disrupting the status quo. As is the case for all government funding decisions the allocation of grant dollars is decided based on data, communication, and pressure from multiple stakeholders. Reprioritization of basic research and substantial, sustained changes in funding must be convincingly justified in the eyes of scientific peers, the public, academic institutions, K-12 educators, industry, and elected officials at all levels, perhaps by adopting strategies successfully employed by patient advocacy groups (e.g. American Cancer Society, American Heart Association). Such a strong, coordinated effort is necessary to effect the broad change in attitude about the value of curiosity driven research using diverse model organisms, and the necessity of reprioritizing basic research support so that it is equal to GMO research subserving applied outcomes.

## Conclusions

Basic research as envisioned by Vannevar Bush, the “…free, untrammeled study of nature, in the directions and by the methods suggested by (the researcher’s) interests, curiosity, and imagination,” comprises a rapidly dwindling proportion of today’s neurobiological and behavioral research literature and funding agency portfolios. Modern NBR is dominated by highly reductionist studies using a miniscule number of species with cutting-edge genetic tools (GMOs), for which a plausible case for biomedical and other human benefits can be made. While the initial surge in GMO research was driven by the community, resources, and novel lines of inquiry delivered by these species, the “runaway selection” observed today is driven by pressure from multiple sources including peers, academic employers, and funding agencies. Founded on the premise of supporting curiosity driven research, the NSF should continue to support basic NBR using EOs (diverse species lacking genetic tools). However, this study finds that EO NBR funding by the NSF is dwindling at an alarming rate. Coupled with declining representation of EOs in the NBR literature, the neuroscience community faces a widening gap in generational knowledge transfer in productive and impactful areas of curiosity driven NBR research using EOs. The reprioritization of curiosity-driven research must not be regarded as an impossibility, but as a necessity.

## Supporting information

S1 Dataset All data used for statistical analyses and figures. (XLSX)

S1 Fig Comparison of non-GMO fish and insect publications from the United States and other countries. A. Curated NBR publications by United States authors (circles) and those outside of the US (squares) using non-GMO insect species B. A. Curated NBR publications by United States authors (circles) and those outside of the US (squares) using non-GMO fish species, Asterisks indicate significant decreases in non-GMO publications from the United States (statistics reported for Figs 4 and 5 in Results and discussion).
(TIF)
