# Supplementary figures and images for: The rise to dominance of genetic model organisms and the decline of curiosity-driven organismal research
Source: PLoS One. 2020 Dec 1;15(12):e0243088. doi: 10.1371/journal.pone.0243088 (PMC7707607; doi:10.1371/journal.pone.0243088)

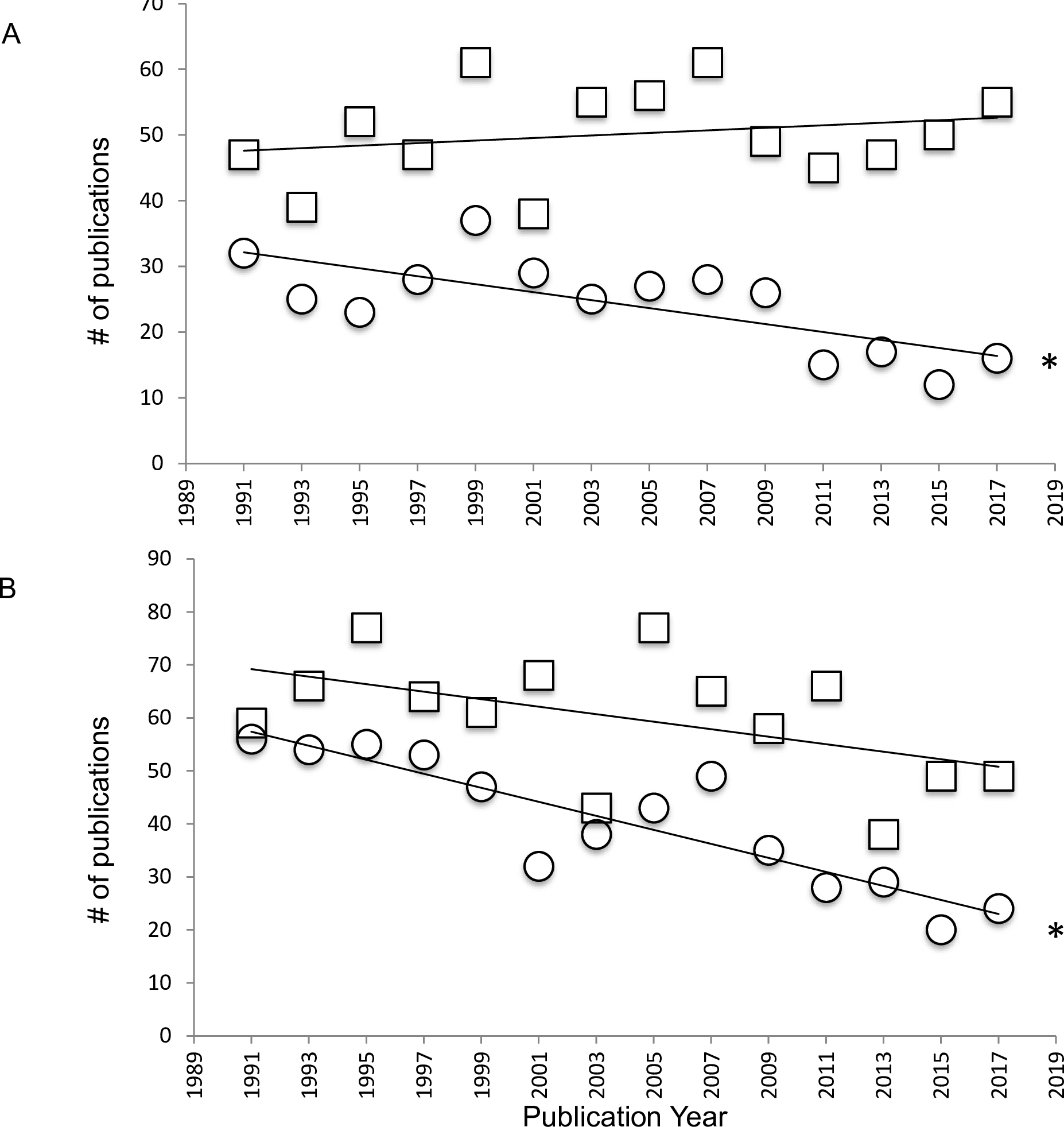

Supplement: S1 Fig — A. Curated NBR publications by United States authors (circles) and those outside of the US (squares) using non-GMO insect species B. A. Curated NBR publications by United States authors (circles) and those outside of the US (squares) using non-GMO fish species, Asterisks indicate significant decreases in non-GMO publications from the United States (statistics reported for Figs 4 and 5 in Results and discussion). (TIF) [file pone.0243088.s002.tif]
